# Effect of Different Black Quinoa Fractions (Seed, Flour and Wet-Milling Coproducts) upon Quality of Meat Patties during Freezing Storage

**DOI:** 10.3390/foods10123080

**Published:** 2021-12-10

**Authors:** Estrella Sayas-Barberá, María Maite Valero-Asencio, Casilda Navarro Rodríguez-Vera, Juana Fernández-López, Claudia Monika Haros, José Ángel Pérez-Álvarez, Manuel Viuda-Martos

**Affiliations:** 1 IPOA Research Group, Agro-Food Technology Department, Centro de Investigación e Innovación Agroalimentaria y Agroambiental (CIAGRO-UMH), Miguel Hernández University, Orihuela, 03312 Alicante, Spain; estrella.sayas@umh.es (E.S.-B.); maria.valero21@goumh.umh (M.M.V.-A.); casilda.navarro@umh.es (C.N.R.-V.); j.fernandez@umh.es (J.F.-L.); ja.perez@umh.es (J.Á.P.-Á.); 2 Cereal Group, Instituto de Agroquímica y Tecnología de Alimentos (IATA-CSIC), 46980 Valencia, Spain; cmharos@iata.csic.es

**Keywords:** patties, freezing storage, quality, quinoa, coproducts, wet milling

## Abstract

In this study, the quality of meat patty samples containing different black quinoa fractions (seed, flour and wet-milling coproducts) was evaluated during freezing preservation. Composition, physicochemical parameters (aw, pH, colour and texture), cooking properties, lipid oxidation and sensory characteristic were studied in four batches (control and 8% concentration of quinoa seed, flour and wet-milling coproducts added) at 30, 60 and 90 days of freezing (−20 ± 1 °C). Different black quinoa fraction addition affected (*p* < 0.05) physiochemical properties, improved cooking properties and reduced lipid oxidations during freezing storage. Batches with flour and wet-milling coproducts added were the most stable for texture parameters and lipid oxidation during freezing. The results obtained showed that quinoa wet-milling co-products could be considered a valuable sustainable and organic food ingredient, maintaining nutritional and global qualities of the fresh meat product. In addition, freezing storage is an effective way to prolong the shelf life of patties with different black quinoa fractions, added without affecting quality.

## 1. Introduction

The agro-food industry, from primary production to distribution, has represented one of the fundamental economic supports and pillars worldwide during different crises. The coronavirus (COVID-19) pandemic is one of them, and thanks to this industry it has been possible to satisfy the food needs of the population. The supply chain has continued to work with the implementation of extra sanitary safety measures, which have led to a significant economic cost for companies, in order to minimize the hazards of the coronavirus, while considering food safety requirements [1]. Moreover, the COVID-19 crisis has highlighted the relationship between people and nature.

The consumer’s requirements have been changing, not only when looking for functional foods or bioactive ingredients to improve their immune system [1], but also for sustainable food consumption and production within the circular economy. On top of this, obtaining new food ingredients or products from food industry by-products and coproducts is seen positively by most people.

Aspects as important as health, ethical and environmental conditions impact innovation and development within the food industry. The recommendations from health organizations for following a diet rich in plant-based foods and reducing the consumption of processed meat have influenced consumer demand. In addition, focusing on sustainable production with zero residue and/or finding processes that allow the valorisation of by-products and co-products, which could be used as new functional ingredients in food, has provided an opportunity for innovation in the food industry. The purpose of current and future technological processes is to contribute to sustainability and adopt conventional technologies in order to guarantee the conservation of natural resources for future generations [2].

In recent decades, research in the agro-food industry has focused on searching for viable alternatives to find a second life or increased utility for a large number of by-products and co-products from industrial activity, which are discarded for different reasons (technological, commercial and/or economic) and, in this way, to contribute to a more sustainable economy [3].

Regarding food industry innovation, the development of heathier food based on scientific evidence on the relationship between diet and health must be mentioned. Besides, many diseases are related to unhealthy food such as diabetes and obesity, among others. Moreover, these should be the main reasons for research in this field by focusing on the development of new products, whose nutritional and functional properties help to reduce most of these risks [4]. Especially noteworthy are different strategies in the development of new healthier meat products focused on decreasing negative components and/or increasing the presence of beneficial ingredients [5]. Within this second strategy, quinoa (*Chenopodium quinoa* W.), a pseudo-cereal belonging to the *Chenopodiaceae* family, as a novel and healthy ingredient, has been used for the reformulation of different foods [6].

Quinoa is considered a very important crop due to its high nutritional quality (14–20 g protein/100 g dry basis and bioavailable amino acid), is suitable for celiac patients, due to the lack of gluten, and furthermore the quinoa grain can be used almost completely [6,7]. The consumption of quinoa has beneficial effects on cardiovascular, metabolic, and gastrointestinal problems [8,9]. Quinoa flour is used to enrich gluten-free products, especially from the bakery, in addition to promoting metabolism due to its rich content of elements that are related to haematological functions and enzymatic co-factors [10,11]. It is reported that quinoa (quinoa paste, dried quinoa paste or quinoa flour) has been incorporated as a functional ingredient and as ingredient replacement in meat preparations [6,12,13,14].

Quinoa shows interesting technological properties and has become an excellent non-meat ingredient to use as a filler, binder and fat replacer in meat products [9]. Quinoa flour has been used to improve burger quality and eliminate certain components, such as soy protein and breadcrumbs, which could provoke some types of allergy [12,13,14]. Shorky [12] replaced 5, 10 and 15 g/100 g of meat with the same concentration of quinoa flour and eliminated soy protein to obtain a soy-free product. Improvements were reported in nutritional and cooking properties in all concentrations studied. In another study on burgers Özer and Seçen [13] eliminated breadcrumbs and incorporated 3, 5, 7 and 10 g/100 g of quinoa flour, reporting no effect on quality properties; however, a decrease in lipid oxidation during frozen storage was obtained. In a similar way, Bagdatli [14] obtained gluten-free beef meatballs, incorporating different quinoa flour levels (up to 7.5%), and showed that the final product presented good acceptability and an improvement in its nutritional value. In addition, quinoa has been used as a replacement for starch and fat in cooked products [9,13], confirming its technological potential.

A wet-milling coproduct is obtained from the processing of quinoa flour, which is considered a functional and healthy ingredient due to its rich fibre content and bioactive (mainly phenolic) compounds [15]. This wet-milling coproduct has become a potential food ingredient contributing to sustainable food production. This product been studied by several authors, including its incorporation into food [15,16,17], but few studies have been made about the effect of different black quinoa fractions on the shelf life of meat products during freezing preservation [13,18]. Enhanced knowledge of the changes in the quality of meat products containing non-meat ingredients during freezing and defrosting is necessary to avoid undesirable changes that may affect acceptance by the final consumer. Other authors have reported that quinoa proteins show good freeze-thaw properties which might be caused mainly by the generation of a gel-like-three-dimensional structure resistant to retrogradation. These properties could be useful for application in a wide range of food products [9].

Therefore, the aim of this work was to study the effect of the incorporation of several black quinoa (*Chenopodium quinoa* W.) fractions (wet-milling coproduct, black quinoa seed and flour) upon chemical composition, physicochemical parameters, cooking properties, lipid oxidation, and sensory properties in meat patties during freezing preservation.

## 2. Materials and Methods

### 2.1. Experimental Design

This study was carried out with black quinoa wet-milling coproduct, as a novel sustainable ingredient, and in addition, other more usual fractions (quinoa seed and flour) were also added for comparison between them. Four batches of a meat patties model system were elaborated: control batch without added quinoa (CB), batch with 8 g/100 of sample of quinoa seed (SQB), batch with 8 g/100 of sample of quinoa flour (FQB) and batch with 8 g/100 of sample of wet-milling coproduct (CoQB). After elaboration, the 4 batches were characterized and stored in freezing at −20 °C ± 1 °C for 90 days. The experiment was replicated three times at the Pilot Plant of the Miguel Hernández University, according to an industrial processing method.

### 2.2. Plant Materials and Preparation of the Different Fractions of Quinoa

Organic black quinoa (*Chenopodium quinoa* W.) seeds were used in the present study, with the following composition: moisture 5.27 g/100 of sample, protein 12.44 g/100 of sample, fat 5.31 g/100 of sample, dietary fibre 18.59 g/100 of sample, starch 55.02 g/100 of sample and ash 2.37 g/100 of sample [19]. The saponin content from seeds was removed by washing treatment (quinoa/water ratio of 1:15), which was maintained at 55 °C for 30 min in agitation [20]. Subsequently, the saponin-free quinoa was dehydrated over 24 h to 60 °C in a mixed oven (Mychef, Distform, Foodservice Technology). To obtain quinoa flour, half of the dried saponin-free quinoa was milled to a particle size <0.6 mm using a laboratory seed mill (Kitchen Aid) for 3 min at 15 °C and the other half of the dried quinoa was used as whole seeds. The black quinoa wet-milling coproduct, from organic farming is considered a fibre-rich fraction and was obtained following the methodology developed by Ballester et al. [21]. This fraction showed the following composition: moisture 8.21 g/100 of sample, protein 15.92 g/100 of sample, fat 3.29 g/100 of sample, dietary fibre 38.30 g/100 of sample, starch 27.72 g/100 of sample and ash 3.13 g/100 of sample [20].

### 2.3. Meat Patties Processing, Freezing Preservation, Thawing and Cooking Conditions

The meat matrix chosen was a meat patties model system (beef meat 100 g/100 of sample, and salt 1.5 g/100 of sample). This basic formulation was used in all batches, as follows: a control batch (CB), only the basic formulation; the other three were formulated using the basic formulation with the addition of different black quinoa fractions, quinoa seed batch (SQB), quinoa flour batch (FQB) and wet-milling coproduct quinoa batch (CoQB), in a concentration of 8%, according to previous studies [20].

The beef meat was ground with a screw mincer through an 8 mm plate (MAINCA PM-98) and mixed with salt (1.5 g/100 of sample) for 3 min until its homogenization. Subsequently, it was divided in four batches and the different quinoa fractions were added at 8 g/100 of sample, except for the control batch. The 4 batches, including control, were mixed again for 3 min. Then, they were moulded in a patty maker to obtain patties of approximately 80 g and 9 cm diameter, assisted by plastic packing films to maintain the shape. Samples from each batch were separated for their chemical, physicochemical and sensory characterization.

Patties from each formulation were vacuum packed in sterile bags, frozen in an industrial freezer and stored at −20 °C ± 1 °C for 90 days. Three samples from each batch at 30, 60 and 90 days were thawed at refrigeration temperature (2–5 °C) overnight to an internal temperature of 2–5 °C and analysed to study the effect of freezing storage time on lipid oxidation, physicochemical parameters, cooking properties and sensory acceptance. pH, aw and colour analysis were performed in raw samples (before cooking), texture parameters after cooking and lipid oxidation, and sensory analysis was evaluated both, before and after cooking. Patties from each batch were cooked at 170 °C in a convection oven up to an inner temperature of 72 °C and cooled to 21 °C for analysis.

### 2.4. Chemical Composition

The moisture, protein, ash and fat content were determined according to the Association of Official Analytical Chemists [22]. All analyses were evaluated in triplicate. The results were expressed as g/100 g product (%).

### 2.5. Physicochemical Properties

pH was analysed using a pH-meter (Crison micro pH meter 2001 model GLP 21, Crison Instrument S.A., Barcelona, Spain), with three readings per sample.

Water activity (aw) was determined with a Novasina hygrometer (SPRINT TH-500, Pfäffikon, Switzerland) in duplicate, at a temperature of 25 ± 2 °C.

The CIE L*a*b* colour space (Lightness: L*; red/green coordinate: a*; yellow/blue coordinate: b*) was analysed using a Minolta CM-700d spectrophotometer (Minolta Camera Co., Osaka, Japan); illuminant D_65_, an observer angle of 10° and SCI Mode were selected. Nine determinations were taken for each sample [23].

Texture profile analysis (TPA) was evaluated in cooked samples using a Texture Analyzer TA-XT2 (Stable Micro System, Surrey, England) (Claus, 1995). The texture analyses were performed upon cubes of 1 cm^3^ from each batch and were subjected to a 2-cycle compression to 70% of the sample length using a cylindrical probe of 7.5 cm at a compression load of 25 kg and a crosshead speed of 1 mm/s at 15–20 °C. The texture parameters (hardness (kg), springiness (mm), adhesiveness (kg × mm), and cohesiveness (dimensionless) were determined according to Bourne [24] using Stable Micro Systems Software for Exponent Texture Analysis.

### 2.6. Lipid Oxidation

The lipid oxidation was evaluated by the 2-thiobarbituric acid (TBA) method following Rosmini et al. [25]. Triplicate samples were analysed for each batch, before and after cooking. Sample extracts (1 g of sample + 0.5 mL TBA solution 0.3 mM + 1.5 mL trichloroacetic solution 10 g/100 mL (*w*/*v*)) were heated for 10 min in a boiling water bath (100 °C) to develop a pink colour, cooled with tap water and centrifugated at 5500 rpm for 25 min in a centrifuge (Alresa HZ50, Orto Alresa, Aljavit, Madrid, Spain), and the absorbance of the supernatant was measured spectrophotometrically at 532 nm using a UV spectrophotometer (Unicam Ltd., Cambridge, UK). The thiobarbituric acid reactive substances (TBARS) were calculated using a 1,1,3,3-tetraethoxypropane standard curve and expressed as mg malonaldehyde (MDA) by kg of product (mg malonaldehyde/kg sample).

### 2.7. Cooking Properties

Cooking properties were determined to study the behaviour of the meat matrix during cooking. Before and after cooking, the weight (g), diameter (cm) and % fat of three patties from each batch were measured during freezing storage.

The following mathematical formulae were used to calculate shrinkage (%) and cooking yield (%):

(1) $$\mathrm{Shrinkage}\;\left(\%\right)=\frac{\left(\mathrm{raw}\;\mathrm{diameter}-\mathrm{cooked}\;\mathrm{diameter}\right)}{\left(\mathrm{raw}\;\mathrm{diameter}\right)}\times 100$$

(2) $$\%\;\mathrm{Cooking}\;\mathrm{yield}=\frac{\left(\mathrm{Cooked}\;\mathrm{patties}\;\mathrm{weight}\right)}{\left(\mathrm{Raw}\;\mathrm{patties}\;\mathrm{weight}\right)}\times 100\;$$

### 2.8. Sensorial Analysis

The sensorial analysis was made by a trained panel (12 judges, the same for each sensorial assay carried out during the storage time), regular consumers of patties. They were trained in the terminology of the analysis and to detect off-odour and -colour, related to patties spoilage [26]. Two trials were performed, one before and the other after cooking (raw and cooked sample), for each time (0, 30, 60 and 90 days) and each batch studied.

Before cooking, samples from each batch, immediately after thawing, were subjected to sensorial analysis where odour and colour were scored using a 10-point scale: 0-No off-odour and 10-Extreme off-odour; and 0-Very light red and 10-Dark red. Furthermore, after cooking, odour, colour and detection of particles were evaluated using the following 10-point scale: 0-None and 10-Extremely intense, for odd odour; 0-Very light red and 10-Dark red; and 0-no particles and 10-abundant particles [26].

In all the analysis sessions, two samples of each batch, identified by 3-digit code and presented in a random order, were presented to each panellist.

### 2.9. Statistical Analysis

The complete process (patties manufacture) was replicated three times (three independent batches). Each replication was completed on a different production day and each batch was examined in triplicate. Statistical analysis was carried out using the program SPSS version 21.0 (IBM, SPSS Statistics Software, Inc., Chicago, IL, USA). Different Analyses of Variance (ANOVA) were applied: one factor (batches) for patties characterization and two factor (batches and storage time) to study the effect of storage time on quality parameters. If statistically significant differences were found, a Tukey-b post-hoc test was performed at 5% significance level (*p* < 0.05).

## 3. Results

### 3.1. Chemical Composition of Meat Patties with Different Black Quinoa Fractions (Seed, Flour and Wet-Milling Coproduct)

The chemical composition of meat patties, freshly prepared (day 0), formulated with different black quinoa fractions (seed, flour and wet-milling coproducts) is shown in Table 1. For ash, protein and fat content, no statistical differences (*p* > 0.05) were found between batches. CB presented the highest moisture content. The addition of quinoa decreased the moisture content in all patties and this effect could be due to an increase in solid contents in the products due to the lower moisture value (5.27%) of the added quinoa (Shokry, 2016). FQB showed the least moisture content (*p* < 0.05). In the same way, other authors have reported the decrease in moisture content in patties when dry ingredients were added: tiger nut fibre [27], oat and wheat fibre [28] and quinoa flour [13]. In the case of quinoa fractions, their addition in sausages also decreased moisture content [15]. The chemical composition of patties with quinoa fractions is in accordance with values reported as usual for this type of meat product [12,20,29]. This result would indicate that quinoa fractions added at 8% could be used as a suitable meat protein replacer, without affecting the overall nutritional quality of the meat product.

### 3.2. Effect of Freezing Storage on Physico-Chemical Parameters of Meat Patties with Different Black Quinoa Fractions

The evolution of pH, water activity (aw) and colour parameters of the different batches are presented in Table 2. The inclusion of black quinoa fractions to the meat matrix did not affect the pH (*p* > 0.05) of samples, and all of them showed similar behaviour during the whole storage time (Table 2). pH values increased during the first 30 days of freezing storage which could be due to proteolytic phenomena that favour the formation of basic compounds (*p* > 0.05) [30]. Other studies reported that quinoa flour [14] and other fibre-rich flours did not influence pH values in meat products [28].

Water activity values (Table 2) showed small significant variations during storage time, which may be due to changes in free water within the meat matrix, because of temperature fluctuations during freezing storage. Holman et al. [31] reported that temperature fluctuations result in more free water released, due to muscle protein denaturation during thawing. There were no significant differences (*p* > 0.05) between samples, all following the same water activity behavior during freezing storage.

Regarding colour (Table 2), changes in meat pigments began during the preparation of patties, especially during chopping and mixing, which increases the surface area exposure to oxygen, leading to oxidation of myoglobin, which continue during the storage. Decreases in the colour stability of meat have been described after freezing and thawing that could be encouraged by denaturation of the globin of the myoglobin, and the formation of free radicals by lipid oxidation, which could support oxidation to metmyoglobin form [32]. Likewise, the incorporation of non-meat ingredients can affect some colour parameters of meat products [12].

In this study, frozen storage and type of quinoa fraction added affected colour stability (*p* < 0.05) (Table 2), which could influence consumer perception. Patty decolouration has been observed during frozen storage and this was dependent on storage temperature. In addition, some interactions between oxidizable meat substances that can occur during frozen storage could also affect colour [33].

Lightness (L*) evolution of patties during storage was dependent on the quinoa fraction added (*p* < 0.05) and also on the storage time (*p* < 0.05) (Table 2). In general, it could be said that the addition of quinoa coproduct or seeds decreased L* values, while the incorporation of quinoa flour increased lightness, compared with control batch (*p* < 0.05). L* depends on other factors such as pH, water retention activity (CRA), fat content and free water, becoming modified by incorporating functional ingredients such as quinoa and its derivatives [34]. In other studies of patties enriched with non-meat ingredients, a decrease in lightness was also described, which could be caused by the formation of gel-type structures between the meat and the non-meat protein [35]. This could explain lightness values in SQB and CoQB patties. The increase in lightness when quinoa was added as flour have been reported in other studies [12,13], which would indicate that the meat matrix with added quinoa flour may be lighter than the other matrices formed.

The a* coordinate is related to the concentration and changes in the oxidation states of haemo-pigments and to lipid oxidation [34]. The quinoa added decreased the a* values, and the lower values were those of the CoQB (Table 2). This result is similar to that found in other studies, which would indicate that the incorporation of different flours into meat matrices produces a diluting effect of the meat pigments [13]. Pellegrini et al. [19] reported a* value of 1.38 for black quinoa flour, which could be via maintaining reducing effect on the meat matrix. During storage, a loss of the red coordinate (a*) was observed for all batches, except for the CoQB. The a* decrease has been observed during the storage of meat products due to the oxidation of myoglobin and/or oxymyoglobin (ferrous iron) and the formation of metmyoglobin (ferric iron) [13,33,36]. The redness of CoQB did not decrease during frozen storage, which would indicate that the black quinoa co-product would be protecting the meat matrix against meat oxidation and stabilizing the red colour during freezing.

Differences in yellowness (b*) were significant between batches and storage times (*p* < 0.05) (Table 2). The addition of quinoa coproducts and seed decreased b* coordinate, while the quinoa flour increased it in comparison to control sample. CoQB presented the lowest values (*p* < 0.05) during the preservation process. The decrease in the value of b* coordinate is attributed to a browning reaction [37]. The b* coordinate is related to the ingredients of the products, the meat matrix and the oxidation of the pigments.

### 3.3. Effect of Freezing Storage on Texture Parameters of Patties with Different Black Quinoa Fractions (Seed, Flour and Wet-Milling Coproducts)

Table 3 shows the texture profile analysis of patties (cooked) during freezing storage at 0, 30, 60 and 90 days. The addition of quinoa affected the texture properties, and this was dependent mainly on the type of quinoa fraction added (*p* < 0.05). CoQB exhibited the highest values for hardness, springiness and chewiness during all storage times, which could be related to the integration of this quinoa coproduct in the meat matrix, which would reinforce the meat structure. On the other hand, this structure would seem not to be significantly cross-linked, because cohesiveness values were the lowest (*p* < 0.05). Several variations in texture properties during storage time have been detected but in most cases, at the end of storage, the values were similar to initial values. On the other hand, when quinoa was added as flour, hardness, cohesiveness and chewiness (*p* < 0.05) of patties decreased, which could be related to the easier and better integration of quinoa flour (with respect to quinoa seeds or coproduct) in the meat matrix.

CoQB exhibited the highest values for hardness, springiness and chewiness during all storage times, which could be related to the integration of this quinoa coproduct into the meat matrix, which would reinforce the meat structure, although this structure would not seem to be significantly cross-linked, because cohesiveness values were the lowest (*p* < 0.05). Several variations in texture properties during storage time have been detected but in most of the cases, at the end of storage, the values were similar to initial values. On the other hand, when quinoa was added as flour, hardness, cohesiveness and chewiness (*p* < 0.05) of patties decreased, which could be related to an easier and better integration of quinoa flour (than quinoa seeds or coproduct) in the meat matrix.

Other studies have reported that texture parameters were affected by the incorporation of non-meat ingredients to the meat product formula, since it could modify the interaction between protein–water and protein-protein, and also by the water and oil binding ability of the fibre-rich ingredient added [38,39].

Changes in texture properties have been reported due to different causes, during freezing, freezing storage and thawing. On the one hand, the harm to structural integrity caused by ice crystals has been reported in meat, and on the other hand in processed meat products [33], would involve other mechanisms, such as the loss of water and protein oxidation [32]. All batches presented changes in texture parameters, except for the batch with co-product, which would indicate that the fibre-rich fraction of quinoa (co-product) would stabilise the texture during freezing, freezing storage and thawing.

### 3.4. Effect of Freezing Storage on Lipid Oxidation of Patties with Different Black Quinoa Fractions (Seed, Flour and Wet-Milling Coproducts)

**Figure 1:**
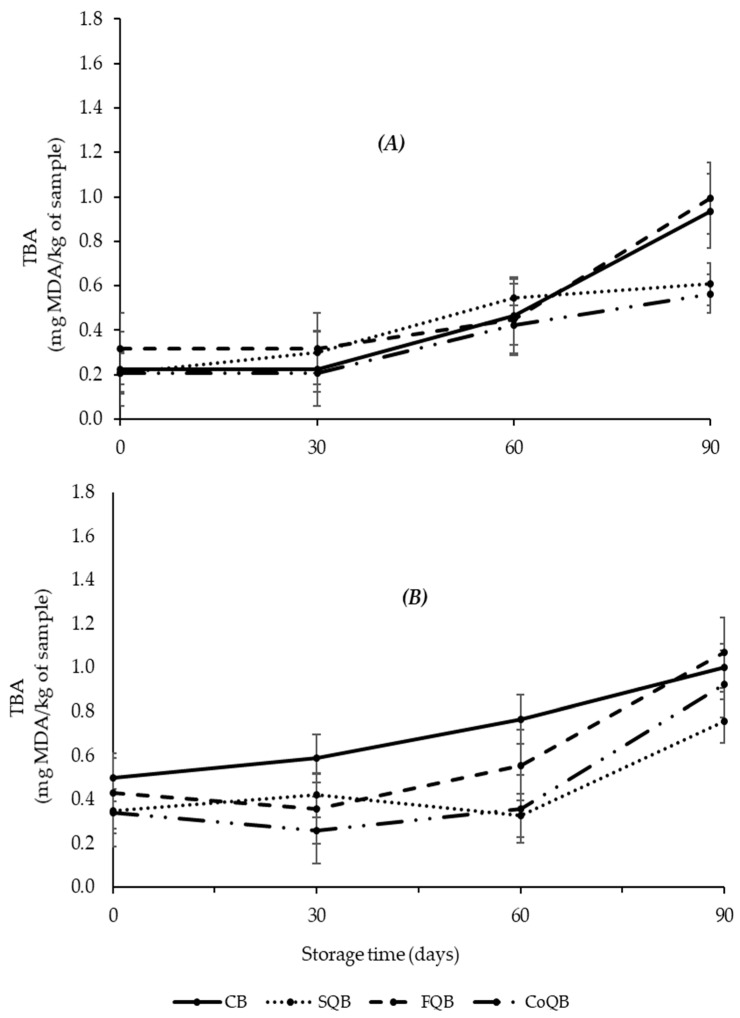
Lipid oxidation of meat patties [raw (**A**) and after cooking (**B**)] formulated with different black quinoa fractions (seed, flour and wet-milling coproducts) during the storage period (90 days). CB: control batch; SQB: batch with 8% of black quinoa seeds; FQB: batch with 8% of black quinoa flour; CoQB: batch with 8% of black quinoa coproducts.

The evolution of lipid oxidation in meat patties before and after cooking during freezing storage time is shown in Figure 1. In raw samples, TBA values increased with freezing storage time in all samples (*p* < 0.05), which would mean that the secondary lipid oxidation continued during freezing storage. This oxidation is recognized as one of the greatest obstacles to the conservation of frozen meat [40]. During freezing storage, some temperature fluctuations can take place, which could induce the formation of extracellular ice crystals, which increases cell disruption, releasing prooxidant compounds and promoting oxidation [41]. In this study, the increase in TBA values was dependent on the type of quinoa fraction added and the storage time (*p* < 0.05). All raw samples showed a similar behavior in TBA evolution during freezing storage time until 60 days (*p* > 0.05), which could indicate that the different black quinoa fractions did not affect the evolution of lipid oxidation during the first 60 days (Figure 1A). However, during the last 30 days of storage, TBA values in CB and FQB patties continued increasing, while patties with black quinoa flour and coproduct (SQB and CoQB) showed similar TBA values than on previous days (60 days), which could indicate that these ingredients would have a protective effect against lipid oxidation in frozen patties during longer storage time (over 90 days).

At the end of storage (90 days), control and black quinoa flour added to patties showed the highest values. However, it is important to note that TBA values did not reach values higher than 2–2.5 mg/kg sample in any of the patties at any storage time. Below these values, it is accepted that there is still no rancidity in meat products, and therefore rancid aromas were not detected [41].

In general, it could be said that cooked samples showed higher TBA values than the corresponding raw ones, although it is true that in some samples at specific storage times (SQB and CoQB at day 60) this does not happen. The increase in TBA values in meat products before and after cooking is due to the oxidation-promoting effect (propagation phase) of heat treatment, which promotes the decomposition of hydroperoxides [41,42]. Other authors have reported the loss of TBARS during cooking due to the involvement of malonaldehyde and other TBARS in further reactions induced by high temperature. These reactions may include the attachment of these lipid oxidation products to meat proteins [33]. After cooking, the behaviour of TBA values during storage time is similar to that observed in raw samples, showing at the end of storage time control and FQB patties with higher values than SQB and CoQB patties. Therefore, quinoa seeds and wet-milling coproducts have a protective effect against oxidation promoted by heat during freezing storage of patties.

### 3.5. Effect of Freezing Storage on Cooking Properties of Meat Patties with Different Black Quinoa Fractions (Seed, Flour and Wet-Milling Coproducts)

Changes in the meat matrix have been reported during cooking of the meat products, which led to water evaporation and fat migration [27,29]. These changes depend on protein functionality and also on non-meat ingredients, which affect water binding, fat adsorption, cooking properties and consumer acceptance of the cooked product.

**Figure 2:**
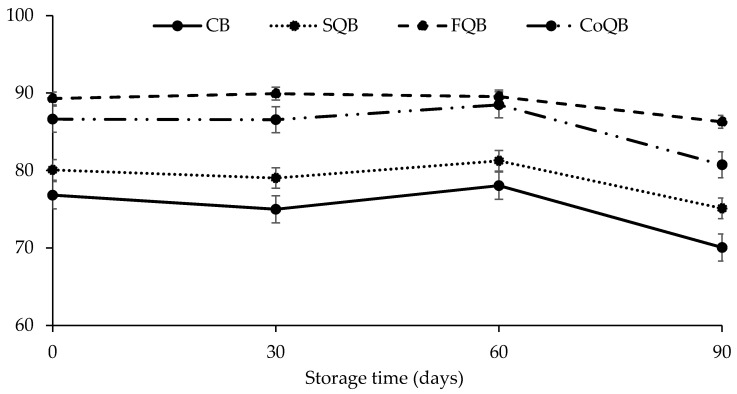
Evolution of cooking yield (%) in meat models with incorporation of different fractions of black quinoa (seed, flour and wet-milling coproducts) during freezing storage (90 days). CB: control batch; SQB: batch with 8% of black quinoa seeds; FQB: batch with 8% of black quinoa flour; CoQB: batch with 8% of black quinoa coproducts.

**Figure 3:**
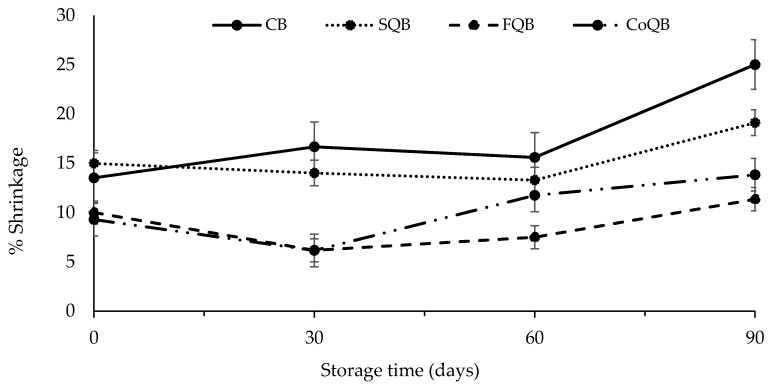
Evolution of shrinkage (%) in patties formulated with different fractions of black quinoa (seed, flour and wet-milling coproducts) during freezing storage (90 days). CB: control batch; SQB: batch with 8% of black quinoa seeds; FQB: batch with 8% of black quinoa flour; CoQB: batch with 8% of black quinoa coproducts.

Figure 2 and Figure 3 show the effect of addition of different fractions of black quinoa to meat patties during freezing storage upon cooking properties (yield and shrinkage, respectively). Both parameters were affected both by storage time and the fraction of quinoa added (*p* < 0.05).

Cooking yield (Figure 2) is one of the most important attributes in evaluation of the cooking behaviour of meat products. Patties with added quinoa flour (FQB) and coproduct CoQB) showed higher yields (*p* < 0.5) (higher than 12% at day 0 or 23% at day 90) than control or patties with added quinoa seeds (SQB), which would indicate a greater retention of water and/or fat during cooking. It would seem that when the whole quinoa grain is added, the interactions between quinoa components and meat matrix are more difficult to carry out than when added as flour or coproduct. This fact could be explained due to the shell and pericarp of grain avoiding this interaction.

During storage, the yield of each batch was not modified until day 60 (*p* > 0.05); during the last 30 days the yield of all samples decreased (*p* < 0.05). Other authors have reported that freezing, frozen storage and thawing would lead to protein oxidation and modification of protein structure that would cause a decrease in the water-holding capacity of meat protein [32,33]. Protein oxidation may be favoured by lipid oxidation, presence of free radicals, and haem pigments, among others. In this study, the increase in lipid oxidation during the last stage of freezing (60–90 days) is in accordance with the decrease in cooking yield in this stage.

The oxidative processes could disturb the protein structure affecting the cooking yield. Recent studies [33] have highlighted the impact of oxidative damage to muscle protein on functional properties, including ability to hold water. They reported that oxidative changes in myofibrillar proteins could cause alterations of water–protein interactions leading to a consequent loss of water holding capacity [34]. Similarly, moisture and cooking yield can be affected by freezing, which could be related to physical damage of the muscle cell structure during frozen storage by the formation of ice crystals [33].

The evolution of shrinkage (%) during frozen storage (Figure 3) showed an inverse behaviour than that observed for % cooking yield. FQB and CoQB patties showed the lowest values for shrinkage during the whole of freezing storage. In this case also, the most significant changes took place during the last 30 days of storage (*p* < 0.05). During cooking (37–75 °C), denaturation and structural changes in the meat proteins have been reported [30,39], inducing a shrinkage of muscle fibres and connective tissue and a decrease in the water holding capacity of the meat, which is perceived by consumers as a decrease in diameter of the meat product (shrinkage).

This phenomenon is often perceived as an undesirable modification of the food [43]. Other studies have reported improvements in cooking properties when quinoa flour was used as fat replacement in burgers [14,44].

These results would indicate that quinoa fractions could be used as frozen protected agents, avoiding significant shrinkage and cooking yield, which could be related to higher water and fat retention in the new meat batter during cooking. In reference to this effect, quinoa flour and wet-milling coproduct fractions could be selected as the better protective agents during freezing.

### 3.6. Effect of Freezing Storage on Sensorial Characteristics of Patties with Different Black Quinoa Fractions (Seed, Flour and Wet-Milling Coproducts)

**Figure 4:**
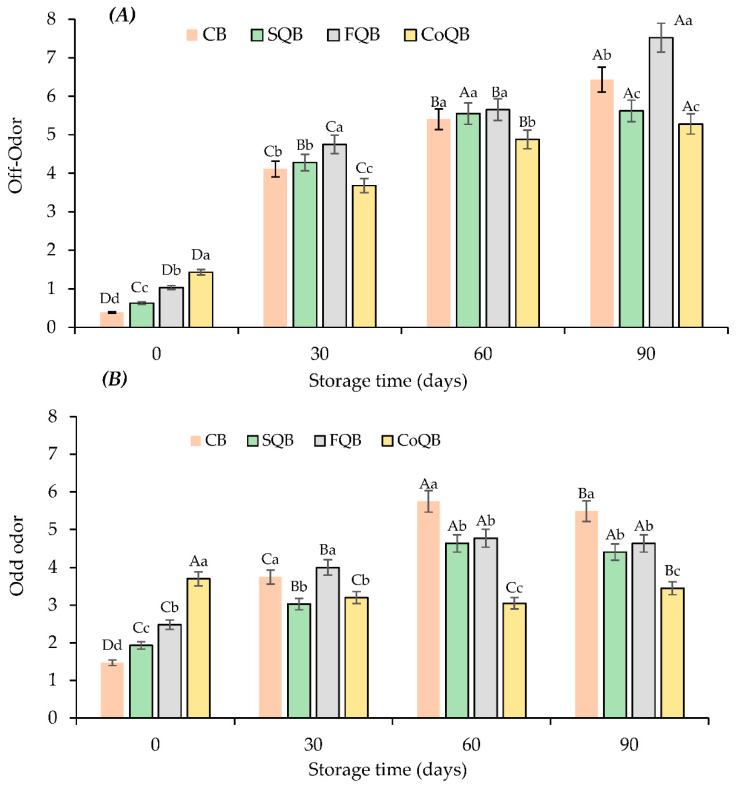
Evolution of off-odour (0-No off-odour and 10-Extreme off-odour) in raw patties (**A**) and odd odour (0-None and 10-Extremely intense) in patties after cooking (**B**) formulated with different fractions of black quinoa (seed, flour and wet-milling coproducts) during freezing storage. CB: control batch; SQB: batch with 8% of black quinoa seeds; FQB: batch with 8% of black quinoa flour; CoQB: batch with 8% of black quinoa coproducts. For each storage time, bars with different small letters indicates the existence of significant differences among samplings (*p* < 0.05). For each sample, bars with different capital letters indicates the existence of significant differences among batches (*p* < 0.05).

**Figure 5:**
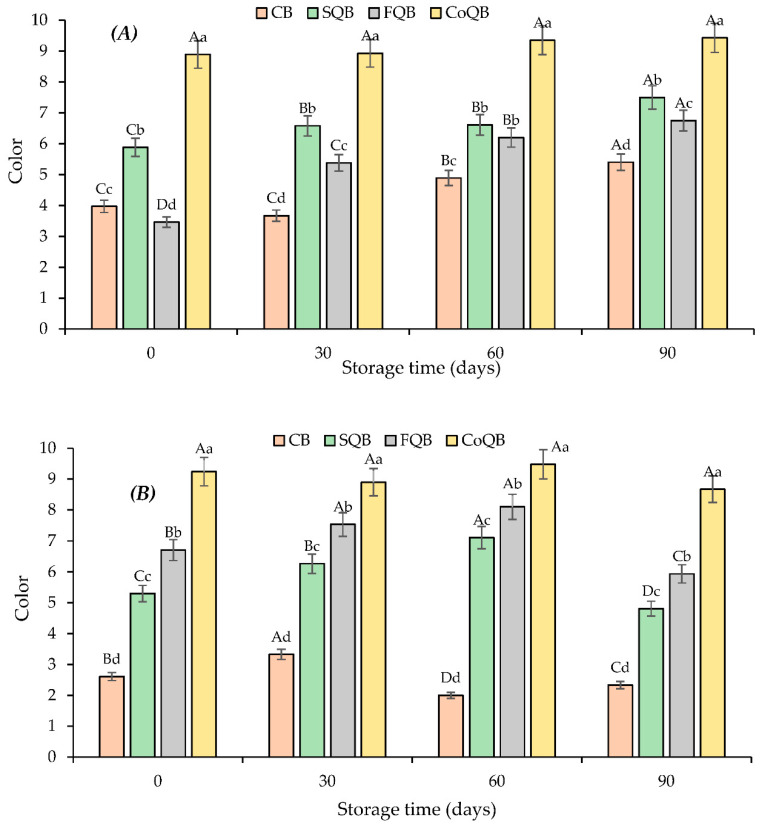
Evolution of sensory colour (0-Very light red and 10-Dark red) in meat patties (raw (**A**) and after cooking (**B**)) formulated with different black quinoa fractions (seed, flour and wet-milling coproducts) during freezing storage (90 days). CB: control batch; SQB: batch with 8% of black quinoa seeds; FQB: batch with 8% of black quinoa flour; CoQB: batch with 8% of black quinoa coproducts. For each storage time, bars with different small letters indicate the existence of significant differences among samplings (*p* < 0.05). For each sample, bars with different capital letters indicate the existence of significant differences among batches (*p* < 0.05).

The sensorial analysis of raw patties (Figure 4 and Figure 5) showed that both parameters evaluated (off-odour and colour) were significantly affected by the storage time and the quinoa fraction added (*p* < 0.05).

At time 0, all raw patties with added quinoa showed higher off-odour scores (*p* < 0.05) than control raw patties, although these off-odours were identified as quinoa odour by panellists (Figure 4). However, changed during storage time, reaching at the end of the storage (90 days) even higher off-odour scores for control patties than quinoa added patties, except when quinoa was added as flour. Freezing preservation increased the unpleasant odor in all samples, as a consequence of oxidative processes (Figure 4A). The behaviour of off-odours in patties was coincident with the result of lipid oxidation (Figure 1A). The sensory analysis of patties after cooking (Figure 4B) determined that CoQB patties presented a higher odd odour score than the rest of the samples at day 0, as also observed in raw patties (Figure 4). During storage, oddodour scores increased for all batches at a value around 5, so the process of freezing and thawing did not adversely affect the product, masking any off-odour in the burgers before cooking. It could be said that quinoa fractions masked the off-odor of raw patties and odd odour of patties after cooking, which had been generated during storage. The sample with the incorporation of co-product presented the highest values for colour (*p* < 0.05) during the storage. Freezing storage and thawing did not affect the colour of the batches after cooking (*p* > 0.05).

In the present study, the addition of non-meat ingredients (quinoa fractions) affected the colour intensity in the final product (Figure 5), as described by other studies [45]. The addition of quinoa increased the colour score in the following order: CB (2.61) < SQB (5.30) < FQB (6.71) < CoQB (9.24).

In general, it could be said that both freezing storage and addition of quinoa fractions increased red colour intensity (Figure 5A), making raw patties darker than control or corresponding patties at time 0, which is well correlated with lightness behaviour shown in the objective colour measure.

Several authors have reported that consumers associate dark colour in meat products with lack of freshness [46] and these colour changes during storage are associated with the state of myoglobin, which showed three natural colours depending on its exposure to oxygen and the chemical state of the iron. The tan or brown colour occurs with the formation of metmyoglobin; in the metmyoglobin state, the iron has oxidized.

Control patties had colour scores at around 5 which means that they were perceived as not too light or too dark by consumers.

**Figure 6:**
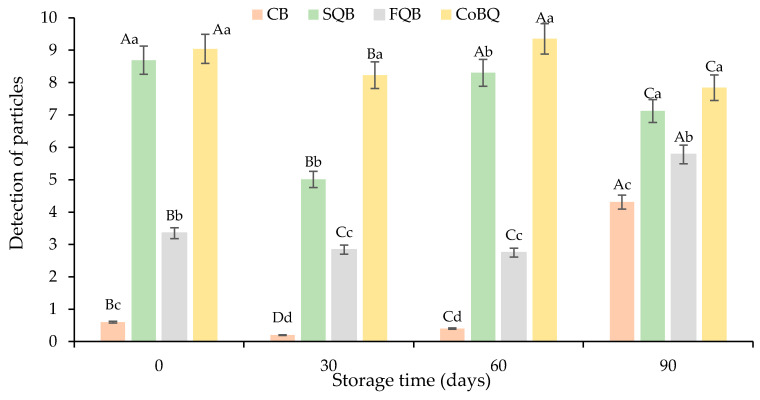
Evolution of scores (sensorial analysis) for detection of particles, in cooked patties formulated with different fractions of black quinoa (seed, flour and wet-milling coproducts) during freezing storage. CB: control batch; SQB: batch with 8% of black quinoa seeds; FQB: batch with 8% of black quinoa flour; CoQB: batch with 8% of black quinoa coproduct. For each storage time, bars with different small letters indicate the existence of significant differences among samplings (*p* < 0.05). For each sample, bars with different capital letters indicate the existence of significant differences among batches (*p* < 0.05).

Regarding the detection of particles (Figure 6), as might be expected, SQB and CoQB patties were scored by panellists (8.69 and 9.04, respectively) higher than control (particles were not detected) or FQB (3.35 patties). These differences were maintained during freezing storage until day 60. At the end of the storage period (day 90), CB and FQB increased their scores (Figure 6), which could be related to the increase in hardness increase in patties during the last month (Table 3).

## 4. Conclusions

The use of different quinoa fractions, especially the co-product, can be considered as an adequate strategy to contribute to the improvement of the nutritional value of meat products and to a more sustainable food production. The incorporation of quinoa presented positive technological effects and did not affect quality during freezing. The addition of black quinoa seeds and coproducts would protect against lipid oxidation in the frozen patties during long storage times (over 90 days). In addition, quinoa seeds and wet-milling coproducts had a protective effect against oxidation promoted by heat, during freezing storage of patties. The lightness, redness and texture parameters in the samples with added quinoa seeds and co-products were not affected during freezing storage.

The results of this study suggest that both quinoa flour and wet-milling coproducts could be used as frozen protected agents in meat patties, avoiding significant quality changes such as shrinkage and cooking losses, changes in texture and colour parameters, while protecting patties from lipid oxidation. Therefore, these quinoa fractions could be used in the meat industry as natural compounds to replace synthetic additives.

## Figures and Tables

**Table 1:** Chemical composition of meat patties formulated with different black quinoa fractions (seed, flour and wet-milling coproducts).

	Samples			
	CB	SQB	FQB	CoQB
**Moisture (%)**	72.92 ± 0.86 ^a^	67.39 ± 1.21 ^b^	64.13 ± 0.49 ^c^	66.88 ± 0.93 ^b^
**Ashes (%)**	2.33 ± 0.26 ^a^	2.51 ± 0.41 ^a^	2.33 ± 0.08 ^a^	2.40 ± 0.17 ^a^
**Proteins (%)**	20.10 ± 0.65 ^a^	20.83 ± 0.79 ^a^	19.80 ± 0.17 ^a^	19.70 ± 0.93 ^a^
**Fat (%)**	3.72 ± 1.01 ^a^	3.28 ± 0.71 ^a^	4.28 ± 0.63 ^a^	3.75 ± 0.40 ^a^

Values are mean ± SD. Values followed by different small letters in the same row indicate significant differences among batches (*p* < 0.05). CB: control batch; SQB: batch with 8% black quinoa seeds; FQB: batch with 8% black quinoa flour; CoQB: batch with 8% black quinoa coproducts.

**Table 2:** Physico-chemical (pH, aw and colour) parameters of meat model systems formulated with different fractions of black quinoa (seed, flour and wet-milling coproducts) during freezing storage (90 days).

Parameters	Batch	Storage Time at −20 °C (Days)			
		Day 0	Day 30	Day 60	Day 90
pH	CB	5.70 ± 0.02 ^Aa^	5.92 ± 0.08 ^Ba^	5.80 ± 0.0 ^Ba^	5.89 ± 0.05 ^Ba^
	SQB	5.63 ± 0.08 ^Aa^	5.88 ± 0.02 ^Ba^	5.85 ± 0.03 ^Ba^	5.86 ± 0.04 ^Ba^
	FQB	5.65 ± 0.05 ^Aa^	5.87 ± 0.05 ^Ba^	5.84 ± 0.03 ^Ba^	5.92 ± 0.02 ^Ba^
	CoQB	5.60 ± 0.03 ^Aa^	5.90 ± 0.02 ^Ba^	5.87 ± 0.04 ^Ba^	5.91 ± 0.04 ^Ba^
Water activity (a_w_)	CB	0.959 ± 0.01 ^Aa^	0.951 ± 0.01 ^Aa^	0.976 ± 0.01 ^Ba^	0.956 ± 0.01 ^Aa^
	SQB	0.963 ± 0.01 ^Ab^	0.953 ± 0.01 ^Aa^	0.973 ± 0.01 ^Ba^	0.959 ± 0.01 ^Aa^
	FQB	0.961 ± 0.01 ^Ab^	0.954 ± 0.01 ^Aa^	0.973 ± 0.01 ^Ba^	0.949 ± 0.01 ^Aa^
	CoQB	0.966 ± 0.01 ^Ab^	0.954 ± 0.01 ^Aa^	0.973 ± 0.01 ^Ba^	0.955 ± 0.01 ^Aa^
Lightness (L*)	CB	37.45 ± 2.09 ^Bb^	36.77 ± 3.69 ^Ab^	35.75 ± 4.74 ^Aa^	35.12 ± 3.57 ^Aa^
	SQB	35.08 ± 1.9 ^Ba^	33.32 ± 3.03 ^Aa^	33.69 ± 2.26 ^Aa^	33.26 ± 2.85 ^Aa^
	FQB	39.17 ± 1.98 ^Ac^	39.51 ± 2.24 ^Ac^	40.38 ± 3.26 ^Ab^	38.81 ± 2.03 ^Ab^
	CoQB	34.44 ± 1.78 ^Aa^	30.70 ± 2.12 ^Aa^	31.63 ± 2.05 ^Aa^	33.77 ± 2.02 ^ABa^
Redness (a*)	CB	7.16 ± 0.98 ^Bc^	7.24± 1.56 ^Bb^	7.85 ± 1.56 ^Bb^	5.27 ± 1.65 ^Ab^
	SQB	5.30 ± 1.25 ^Bb^	6.03 ± 1.98 ^Bb^	5.38 ± 1.46 ^Bb^	2.52 ± 0.83 ^Aa^
	FQB	5.79 ± 1.65 ^Ab^	5.42 ± 0.85 ^bA^	4.65 ± 0.98 ^Ab^	4.84 ± 1.41 ^Ab^
	CoQB	2.88 ± 1.00 ^Aa^	3.91 ± 0.66 ^Aa^	3.2 ± 0.65 ^Aa^	4.85 ± 1.67 ^Bb^
Yellowness (b*)	CB	5.72 ± 1.03 ^Abc^	7.49 ± 1.41 ^Bb^	8.98 ± 1.34 ^Bc^	5.51 ± 1.55 ^Aa^
	SQB	4.65 ± 0.84 ^Aab^	7.61 ± 1.08 ^Bb^	7.45 ± 2.01 ^Bb^	4.48 ± 1.88 ^Aa^
	FQB	6.63 ± 0.96 ^Ac^	9.28 ± 1.05 ^Bc^	9.02 ± 1.44 ^Bd^	7.41 ± 1.23 ^Ab^
	CoQB	3.25 ± 1.25 ^Aa^	5.73 ± 0.70 ^Ba^	5.31 ± 1.29 ^Ba^	5.83 ± 2.00 ^Ba^

Values are mean ± standard deviation. Values followed by different capital letters in the same row indicates the existence of significant differences among samplings (*p* < 0.05), and different small letters in the same columns indicate the existence of significant differences among batches (*p* < 0.05). CB: control batch; SQB: batch with 8% of black quinoa seeds; FQB: batch with 8% of black quinoa flour; CoQB: batch with 8% of black quinoa coproduct.

**Table 3:** Texture profile analysis of cooked meat model systems formulated with different fractions of black quinoa (seed, flour and wet-milling coproducts) during the freezing storage (90 days).

Parameters	Sample	Storage Time at −20 °C (Days)			
		Day 0	Day 30	Day 60	Day 90
Hardness	CB	6.72 ± 2.34 ^Ba^	3.57 ± 1.24 ^Aa^	4.367 ± 1.46 ^Aa^	6.85 ± 2.01 ^Bb^
(kg)	SQB	8.99 ± 0.66 ^Bb^	4.78 ± 1.12 ^Aa^	7.96 ± 0.30 ^Bb^	8.95 ± 1.62 ^Bb^
	FQB	5.79 ± 1.11 ^Ba^	4.05 ± 0.72 ^Aa^	6.22 ± 1.32 ^Bb^	4.77± 0.65 ^ABa^
	CoQB	13.63 ± 1.7 ^Ab^	11.50 ± 1.60 ^Ab^	12.94 ±1.11 ^Ac^	12.19 ±2.94 ^Ac^
Springiness	CB	0.28 ± 0.03 ^Ba^	0.21 ± 0.04 ^Aa^	0.30 ± 0.06 ^Ba^	0.33 ± 0.06 ^Ba^
(mm)	SQB	0.29 ± 0.03 ^Ba^	0.22 ± 0.04 ^Aa^	0.29 ± 0.04 ^Ba^	0.33 ± 0.04 ^Ba^
	FQB	0.29 ± 0.04 ^Aa^	0.30 ± 0.06 ^Ab^	0.36 ± 0.03 ^Ab^	0.36 ± 0.03 ^Aa^
	CoQB	0.35 ± 0.04 ^Ab^	0.38 ± 0.05 ^Ab^	0.39 ± 0.04 ^Ab^	0.41 ± 0.06 ^Ab^
Cohesiveness	CB	0.75 ± 0.06 ^Cd^	0.77 ± 0.04 ^Cc^	0.70 ± 0.03 ^Bc^	0.64 ± 0.06 ^Ac^
	SQB	0.68 ± 0.04 ^Bc^	0.73 ± 0.03 ^Cc^	0.68 ± 0.03 ^Bc^	0.61 ± 0.03 ^Ac^
	FQB	0.40 ± 0.03 ^Aa^	0.38 ± 0.03 ^Aa^	0.36 ± 0.05 ^Aa^	0.36 ± 0.05 ^Aa^
	CoQB	0.58 ± 0.06 ^Ab^	0.54 ± 0.05 ^Ab^	0.56 ± 0.03 ^Ab^	0.58 ± 0.05 ^Ab^
Chewiness	CB	1.42 ± 0.57 ^Bb^	0.61 ± 0.27 ^Ab^	0.92 ± 0.38 ^Aa^	1.50 ± 0.47 ^Bb^
(kg)	SQB	1.76 ± 0.56 ^Bb^	0.77 ± 0.22 ^Ab^	1.58 ± 0.37 ^Bb^	1.79 ± 0.16 ^Bb^
	FQB	0.66 ± 0.21 ^Aa^	0.48 ± 0.16 ^Aa^	0.84 ± 0.33 ^Aa^	0.63 ± 0.16 ^Aa^
	CoQB	3.05 ± 0.56 ^Ac^	2.40 ± 0.45 ^Ac^	2.79 ± 0.34 ^Ac^	2.89 ± 0.26 ^Ac^

Values are mean ± standard deviation. Values followed by different capital letters in the same row indicate the existence of significant differences among samplings (*p* < 0.05), and different small letters in the same columns indicate the existence of significant differences among batches (*p* < 0.05). CB: control batch; SQB: batch with 8% of black quinoa seeds; FQB: batch with 8% of black quinoa flour; CoQB: batch with 8% of black quinoa coproducts.

## Data Availability

The data presented in this study are available on request from the corresponding author.
